# Effect of weight-bearing Liuzijue Qigong on cardiopulmonary function

**DOI:** 10.1097/MD.0000000000033097

**Published:** 2023-02-22

**Authors:** Desheng Li, Mei Shen, Xiaoyan Yang, Desheng Chen, Chunxiu Zhou, Qiuyang Qian

**Affiliations:** a Rehabilitation Medicine Department, Shenzhen Longhua District People’s Hospital, Shenzhen City, China.

**Keywords:** cardiopulmonary, diaphragm, exercise, Liuzijue Qigong, non-invasive cardiac output measurement

## Abstract

**Background:**

Since the outbreak of coronavirus disease 2019, many people have had to reduce their outdoor activities. Therefore, a convenient, simple, at-home training method to improve or maintain cardiopulmonary function is required. This study aimed to explore the therapeutic effect of weight-bearing Liuzijue Qigong on cardiopulmonary function in healthy volunteers.

**Methods:**

This study was a longitudinal trial. The health participants completed a 4-week Liuzijue Qigong exercise with 0.25 kg sandbag wore on each wrist. Each training session took 30 minutes to complete 2 consecutive cycles, and 5 times a week. The cardiopulmonary function of participants was evaluated at baseline (T0) and the end of the intervention (T4). Outcomes measures were pulmonary function, diaphragm movement, and cardiac hemodynamic parameters. Paired *t* test was used to analyze differences within the group.

**Results:**

After 4 weeks of weight-bearing Liuzijue Qigong exercise intervention, the differences in the forced expiratory volume in the 1st second (*P* = .006), forced vital capacity rate of 1 second (*P* = .003), maximal mid-expiratory flow curve (*P* = .002), forced expiratory flow at 50% of forced vital capacity (*P* = .003), and maximum ventilatory volume (*P* < .001) of the participants were statistically significant. The diaphragmatic excursion (*P* = .009) under the calm breathing mode and the diaphragmatic contraction speed (*P* = .003) under the deep breathing mode improved significantly. The cardiac output (*P* = .04), cardiac index (*P* = .035), contractility index (*P* = .018), early diastolic filling ratio (*P* = .042), systemic vascular resistance index (*P* = .019), systemic vascular resistance (*P* = .017), and estimated ejection fraction (*P* = .016) of participants improved significantly in the resting stage. At the end stage of fast walking, that is, the sixth minute of six-minute walk test, the stroke volume index (*P* = .048), heart rate (*P* = .019), cardiac output (*P* = .008), cardiac index (*P* = .003), and left cardiac work index (*P* = .028) of participants were significantly increased compared with those before training, and the systemic vascular resistance index (*P* = .003) and systemic vascular resistance (*P* = .005) were decreased.

**Conclusion:**

Weight-bearing Liuzijue Qigong training significantly improved cardiopulmonary function in healthy volunteers, thus representing home-based cardiopulmonary rehabilitation training.

## 1. Introduction

Liuzijue Qigong is a traditional Chinese Qigong, a fitness method focusing on breathing control. Its essence is the integration of breathing control and body aerobic movement training. Liuzijue Qigong pays attention to the pronunciation of Xu, He, Hu, Si, Chui, and Xi when exhaling, accompanied by relaxing, slow, and gentle movements.^[1]^ Compared with conventional exercise training, Liuzijue Qigong exercise is characterized by the interplay between symmetrical physical postures and movements, breathing control, a meditative state of mind, and harmonious mental focus. It has the characteristics of internal and external meditation, a combination of movement and static, and unity of the body and spirit.^[2,3]^ It can regulate the body, breathing, and the mind.^[4]^ Liuzijue Qigong’s action is gentle and slow, does not require a large space, and is a safe, convenient, and simple home training method.

Studies have shown that appropriate sports activities correlate positively with cardiopulmonary health. Aerobic and breathing training are beneficial to maintain heart and pulmonary function.^[5,6]^ Active outdoor activities are advocated. However, since the outbreak of coronavirus disease 2019 (COVID-19), many people have had to reduce their outdoor activities, which might bring challenges to their cardiopulmonary function. People need a convenient, simple, and at-home training method to improve or maintain their cardiopulmonary function.

Most studies of Liuzijue Qigong have shown that it has a positive effect on the breathing and exercise ability of patients with chronic diseases. Studies have shown that Liuzijue Qigong can improve lung function, dyspnea symptoms, exercise endurance, and quality of life in patients with stable chronic obstructive pulmonary disease (COPD).^[1,7,8]^ In addition, Liuzijue Qigong exercise can improve exercise capacity in patients with chronic heart failure,^[9]^ reduce the resting systolic blood pressure in patients with hypertension, and improve the quality of life of participants.^[10]^

Although some studies have evaluated the application of Liuzijue Qigong in patients with chronic diseases, it remains unclear whether Liuzijue Qigong is beneficial when administered as an adjunct to modern rehabilitation in healthy people. We suspect that it could improve ventilation function and enhance cardiopulmonary function in healthy volunteers. Therefore, this study aimed to investigate the effects of Liuzijue Qigong on cardiopulmonary fitness in healthy volunteers.

## 2. Methods

### 2.1. Trial design and oversight

The study was a longitudinal trial to determine the therapeutic effects of Liuzijue Qigong on cardiopulmonary function. The Ethics Committee of Shenzhen Longhua District People’s Hospital reviewed this study in accordance with the principles of voluntariness, fairness, and confidentiality (Ethical review number: 2021112). Before the intervention, we explained the detailed purpose, plan, intervention time, benefits, and potential risks of this study to the participants. Strictly abiding by the voluntary principle, the participants agreed to and signed the informed consent form before entering the study. We strictly enforced termination standards to ensure patient safety. In the course of the intervention, participants also had the right to opt out of the study according to their circumstances. The personal information and related records of participants were kept strictly confidential. The study was registered in the Chinese Clinical Trial Registry (Clinical Trial Registration number: ChiCTR2100047079) in 2021. Any amendment to the protocol required further approval from the Ethics Committee.

### 2.2. Participants

The study was conducted at the People’s Hospital of Longhua District, Shenzhen, from June10, 2021 to August 30, 2021. Participants were recruited from the Outpatient Department of Rehabilitation Medicine, People’s Hospital of Longhua District, Shenzhen by clinicians.

Participants were included if they satisfied the following criteria: they had no cardiopulmonary-related diseases or diseases that might affect cardiopulmonary function in the past; they had not engaged in any regular exercise (at least twice a week) within 6 months before the intervention; they were between 18 and 50 years old; they could complete 30 minutes of moderate-intensity training; they had no limitation of movement because of limb or joint movement obstacles; and they agreed to and signed the informed consent form. Participants with a history of cardiovascular or respiratory diseases, congenital chest malformations and rib fractures, thoracic and abdominal surgery, and other neurological diseases that might affect the respiratory muscles were excluded.

### 2.3. Intervention

Guidance was provided for the participants 3 times a week for 2 weeks before the intervention by an experienced rehabilitation therapist to ensure that each participant fully mastered the method of Liuzijue Qigong training. Participants completed the training 5 times a week in a quiet exercise room for 4 weeks. Each training session took 30 minutes to complete 2 consecutive cycles of Liuzijue Qigong training. During training, the participant wore a 0.25 kg sandbag on each wrist and the exercises were performed according to the Liuzijue Qigong teaching catalog.^[11]^ The general method is to inhale slowly through the nose and exhale through the mouth, with corresponding synchronous body movements, following the lead therapist for rhythmic breathing training. When training, the body should remain relaxed, keeping quiet and maintaining a relaxed mood. In addition to the preparatory posture, the initial posture, and the final posture, Liuzijue Qigong training includes 6 main training movements. The order of breathing and pronunciation is “ Xu, He, Hu, Si, Chui, Xi.” Each breath, pronunciation, and action was repeated 6 times, and then continued to complete the next breathing and pronunciation action (Table 1). All breathing, pronunciation, and actions were carried out in order and recorded as a complete cycle of Liuzijue Qigong training.

**Table 1:** The intervention program.

Intensity	Frequency	Mode	Duration
A 0.25 kg sandbag wore in each wrist	Five times a week for 4 wk	Inhale slowly through the nose and exhale through the mouth, with corresponding synchronous body movements, following the lead therapist for rhythmic breathing training. When training, the body should remain relaxed, keeping quiet and maintaining a relaxed mood	Each training session took 30 min

Pronunciation, mouth shape, and breathing are the unique and core practice methods of Liuzijue Qigong. The basic requirements for training are an accurate mouth shape, pronunciation, and breathing, which are coordinated with the body movements; the body is regarded as a whole, and rhythmic training is carried out.

### 2.4. Outcome measures

The outcome measures are pulmonary function, the function of diaphragm movement, and cardiac hemodynamic parameters. Baseline data and the outcome measures at baseline (T0) were collected before the intervention and the outcome measures were collected again within 1 week after the intervention (T4).

#### 2.4.1. Pulmonary function.

The participant takes a standing position for the test. Pulmonary function data were collected using a digital spirometer (Chest graph HI-101, Japan).^[12]^ The participant rested for 5 minutes after the test and then the measurements were repeated. To obtain acceptable and reproducible criteria, all participants underwent traditional spirometry following the Spanish Society of Pulmonology and Thoracic Surgery requirements.^[13]^ To reduce the error caused by the test, we obtained 3 acceptable measurement results and the average value was taken as the final result. The outcomes including vital capacity (VC), forced vital capacity (FVC), forced expiratory volume in the 1st second (FEV1), FEV1/FVC, maximal mid-expiratory flow (MMEF), forced expiratory flow at 25%, 50%, 75% of FVC (FEF25%, FEF50%, and FEF75%) and maximum ventilatory volume (MVV).

#### 2.4.2. Diaphragm movement.

**Figure 1.**
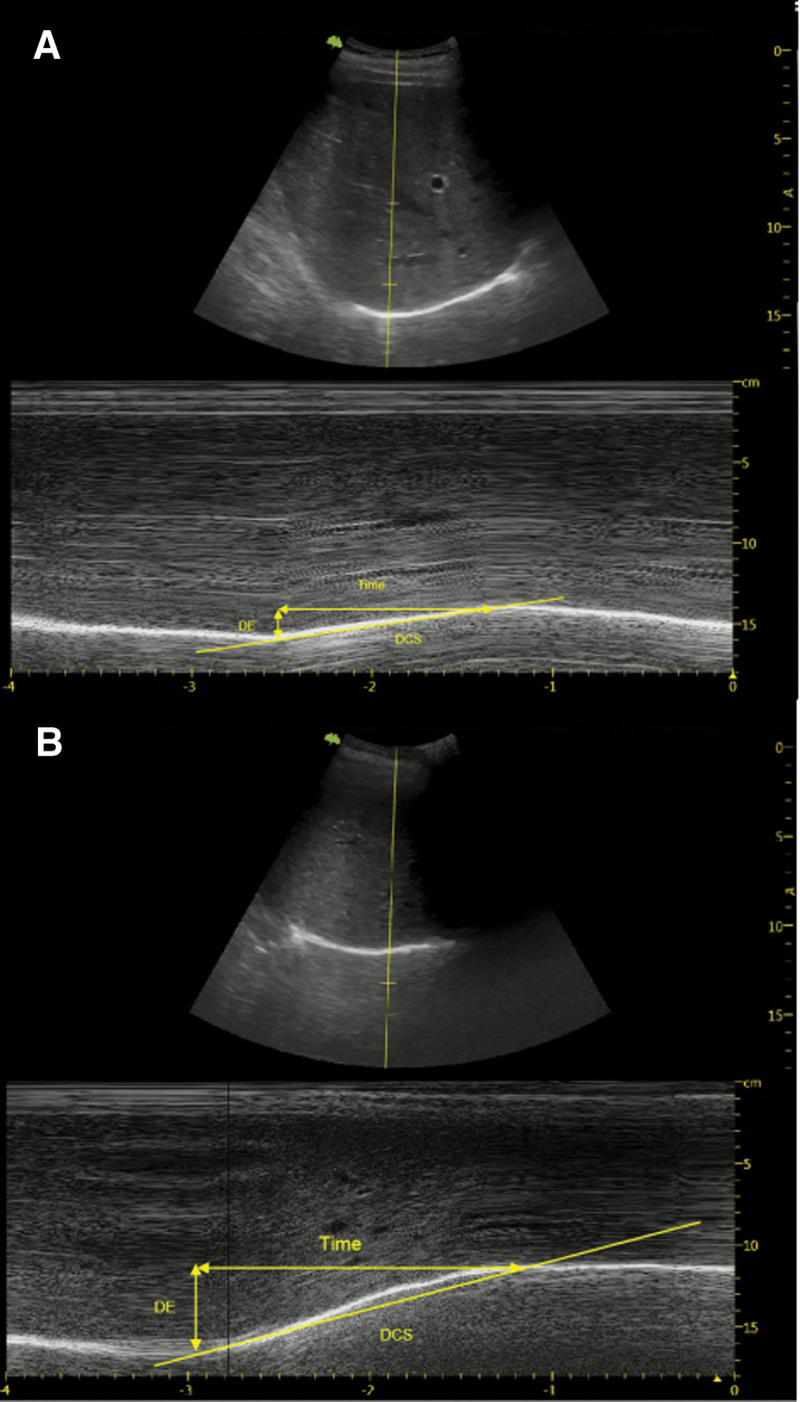
Measurements were performed during calm breathing (A) and deep breathing (B).

M-mode ultrasound examination of the anterior subcostal region measures the athletic capacity of the diaphragm. Two experienced ultrasonic physicians conducted ultrasonographic examinations using a commercially available Doppler echocardiography (Vinno5, Soochow, Zhejiang Province, China) connected to a 2.5 to 3.5 MHz transducer array. Participants were investigated in the morning, 2 hours after a light meal.^[14]^ Ultrasonographic examinations were performed in the supine position. The transducer was placed in the anterior subcostal region between the anterior axillary and the midclavicular lines for the right hemidiaphragm assessment.^[15]^ Participants breathed quietly for a while (>5 cycles) during the test and then performed 3 cycles of deep breathing. In this process, the diaphragmatic excursion (DE) during calm breathing and deep breathing was measured. The diaphragmatic contraction speed (DCS) was measured as the slope (cm/s) from baseline (at the onset of inspiration) to peak DE (Fig. 1). Assessment of DE is an alternative diaphragm function analysis method and is relatively straightforward to perform, with good reproducibility.^[16,17]^

#### 2.4.3. Cardiac hemodynamic measurements.

The six-minute walk test (6MWT) was conducted in a 30-m hospital corridor, following the standard protocol recommended by the American Thoracic Society.^[18]^ The cardiac hemodynamic parameters were determined using Impedance cardiography (ICG) (PhysioFlow^®^ PF07 Enduro™, Paris, France) method. This commercial monitoring system provides continuous noninvasive cardiac output monitoring. Transthoracic bioimpedance is a technique that quantifies the mechanical instead of the electrical activity of the heart. A fundamental principle of transthoracic bioimpedance is that it uses the direct measurement of baseline impedance, ventricular ejection time, heart rate, and the early diastolic filling ratio. These parameters are measured and used to compute other cardiac hemodynamic parameters.^[19]^ It is a noninvasive technology that uses real-time wireless monitoring of morphological-based impedance electrocardiogram signals through a Bluetooth USB adapter. Transthoracic bioimpedance measures cardiac hemodynamic parameters at 1-second intervals, and can provide basic indicators of cardiovascular function, including the stroke volume (SV), stroke volume index (SVi), heart rate (HR), cardiac output (CO), cardiac index (CI), contractility index (CTI), early diastolic filling ratio (EDFR), ventricular ejection time systemic, systemic vascular resistance (SVR), systemic vascular resistance index (SVRi), end-diastolic volume, and estimated ejection fraction (EFest). A previous study showed that in the 6MWT, ICG provides reliable, detailed, and noninvasive cardiac hemodynamic data.^[20]^

Participants were asked to rest in their supine position for 10 minutes before and after 6MWT after connecting the ICG Bluetooth adapter during the data collection process. Cardiac hemodynamic parameters were recorded at 1-second intervals using ICG during 6MWT. Systolic and diastolic blood pressure (SBP and DBP) were measured using the OMRON electronic blood pressure monitor (U30, Dalian, China).^[21]^ The rate of perceived exertion at the end of each test was recorded using the modified 0 to 10 Borg Scale.^[22]^

We divided the whole experiment into 2 stages, that is, the resting state and the walking state. We performed analysis of subject data in the last 5 minutes in resting state and after the 6th minute of fast walking.

### 2.5. sample size

The sample size was calculated based on the FEV1 after 4 weeks of training. A 1-sample design was used, with an assumption of α = 0.05 and 80% power. The sample size was calculated based on the mean ± standard deviation of the FEV1 test results [FEV1: control group 1.34 ± 0.42; Liuzijue exercise group 1.66 ± 0.70].^[2]^ The expected sample size was n = 11.

### 2.6. Blinding

An experienced rehabilitation therapist collected the study data, and he/she did not know the specific method and operation of the study.

### 2.7. Statistical analysis

The study data were analyzed using SPSS (version 26.0; IBM Corporation, Armonk, NY). The normality of the data and the homogeneity of the variance were tested before performing the statistical analysis. Continuous variables are expressed as the mean (standard deviation), and categorical variables are expressed as the frequency (%). Descriptive statistics were used to summarize all participants’ demographic data and clinical variables. For continuous variables that met the normality and homogeneity of variance tests, a paired *t* test was used to analyze differences within the group. *P* < .05 was considered significant.

## 3. Results

**Figure 2.**
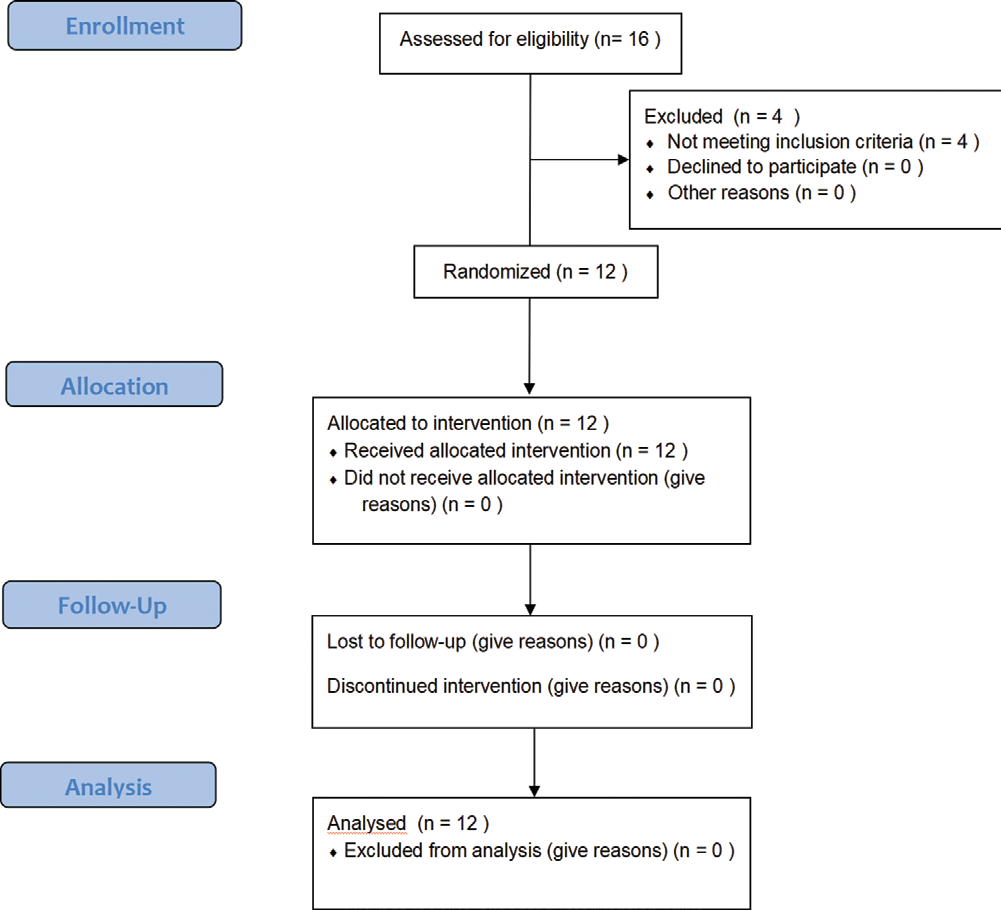
The workflow of the study.

Figure 2 shows the flow of participants throughout the study. A total of 12 participants participated in the trial, all of whom were recruited from the Rehabilitation Medicine Department of the People’s Hospital of Longhua District, Shenzhen, from June 2021 to August 2021. No participant felt unwell or withdrew throughout the whole experiment. The characteristics of the participants are shown in Table 2.

**Table 2:** Baseline characteristics.

	LQG (n = 12)
Sex, n (male:female)	7:5
Age (yr), mean (SD)	35.25 (9.02)
Weight (kg), mean (SD)	68.58 (17.14)
Height (cm), mean (SD)	164.92 (9.40)
Body mass index (kg/m^2^), mean (SD)	24.89 (3.86)

SD = standard deviation.

The results of the pulmonary function tests are shown in Table 3. TheFEV1 (*P* = .006), FEV1/FVC(*P* = .003), MMEF (*P* = .002), FEF50% (*P* = .003), and MVV (*P* < .001) of the participants increased significantly after training. There were no significant differences for VC and FVC.

**Table 3:** The outcome measures of pulmonary function and diaphragm movement in the study.

	T0	T4	*P*
VC (L)	3.75 (0.84)	3.74 (0.74)	.962
FVC (L)	3.49 (0.91)	3.67 (0.76)	.167
FEV1 (L)	2.80 (0.64)	3.19 (0.60)	***.006***
FEV1/FVC	80.83 (7.98)	87.20 (5.80)	***.003***
MMEF (L)	3.08 (0.79)	3.77 (0.95)	***.002***
FEF25% (L/s)	5.49 (1.67)	5.70 (1.77)	.738
FEF50% (L/s)	3.71 (0.94)	4.70 (1.37)	***.003***
FEF75% (L/s)	1.65 (0.76)	1.76 (0.41)	.544
MVV (L)	80.58 (22.24)	98.23 (22.64)	***<.001***
DE (cm)			
Calm breathing	2.20 (0.53)	2.96 (0.68)	***.009***
Deep breathing	4.95 (0.83)	5.44 (1.17)	.118
DCS (cm/s)			
Calm breathing	1.22 (0.40)	1.49 (0.18)	.069
Deep breathing	1.67 (0.77)	2.34 (0.76)	***.003***

Data are presented as mean (SD). Significant results are indicated in bold italic.

DCS = diaphragmatic contraction speed, DE = diaphragmatic excursion, FEF25% = forced expiratory flow at 25% of FVC, FEF50% = forced expiratory flow at 50% of FVC, FEF75% = forced expiratory flow at 75% of FVC, FEV1 = forced expiratory volume in 1st second, FEV1% = forced vital capacity rate of 1 second, FVC = forced vital capacity, MMEF = maximal mid-expiratory flow curve, MVV = maximum ventilatory volume, SD = standard deviation, VC = vital capacity.

The parameters that evaluated the function of diaphragm movement are also shown in Table 3. After training, only the DE (*P* = .009) under the calm breathing mode and the DCS (*P* = .003) under the deep breathing mode of the participants had increased significantly.

After weight-bearing Liuzijue Qigong training, the walking distance in 6MWT (*P* = .032) of participants increased significantly. We tested the cardiac hemodynamic parameters of the participants in the last 5 minutes in the resting state and at the 6th minute in the fast-walking state (Table 4). In the resting stage, the CO (*P* = .04), CI (*P* = .035), CTI (*P* = .018), EDFR (*P* = .042), SVRi (*P* = .019), SVR (*P* = .017), and EFest (*P* = .016) of the participants had improved significantly. At the end stage of fast walking, that is, the sixth minute of 6MWT, the SVi (*P* = .048), HR (*P* = .019), CO (*P* = .008), CI (*P* = .003), and left cardiac work index (*P* = .028) of the participants had increased significantly with those before training, and the SVRi (*P* = .003) and SVR (*P* = .005) had decreased significantly.

**Table 4:** The cardiac hemodynamic parameters during 6MWT.

	T0	T4	*P*
6MWT (m)	597.88 (42.84)	634.13 (42.74)	***.032***
Resting stage			
SV (mL)	80.79 (18.88)	87.45 (21.36)	.133
SVi (mL/m²)	44.64 (6.50)	47.49 (5.37)	.164
HR (bpm)	76.81 (12.96)	76.83 (11.03)	.992
CO (l/min)	6.22 (1.77)	7.08 (1.86)	***.04***
CI (l/min/m²)	3.15 (0.93)	3.76 (0.82)	***.035***
CTI	213.74 (89.13)	238.64 (82.10)	***.018***
VET (ms)	322.17 (42.81)	326.51 (39.83)	.311
EDFR (%)	56.44 (14.21)	61.93 (10.62)	***.042***
LCWi (kg.m/m^2^)	3.95 (1.19)	4.22 (1.11)	.411
SVRi (dyn.s/cm^5^.m^2^)	2164.94 (463.73)	1916.18 (365.57)	***.019***
SVR (dyn.s/cm^5^)	1214.07 (263.68)	1084.28 (212.45)	***.017***
EDV (mL)	128.17 (38.95)	130.60 (36.22)	.641
EFest (%)	65.26 (10.83)	69.96 (11.18)	***.016***
Exercise stage			
SV (mL)	120.26 (29.30)	124.69 (29.07)	.101
SVi (mL/m^2^)	64.82 (9.65)	68.63 (6.77)	***.048***
HR (bpm)	143.27 (19.93)	153.70 (20.60)	***.019***
CO (l/min)	17.28 (4.94)	20.43 (4.57)	***.008***
CI (l/min/m^2^)	9.54 (2.77)	11.49 (2.89)	***.003***
CTI	387.97 (82.36)	397.52 (92.82)	.62
VET (ms)	191.55 (39.82)	187.15 (37.62)	.616
EDFR (%)	78.95 (9.51)	81.95 (16.23)	.377
LCWi (kg.m/m^2^)	11.59 (3.82)	12.90 (4.34)	***.028***
SVRi (dyn.s/cm^5^.m^2^)	817.88 (148.82)	706.71 (97.55)	***.003***
SVR (dyn.s/cm^5^)	459.04 (81.42)	399.04 (56.05)	***.005***
EDV (mL)	167.82 (52.32)	167.02 (48.64)	.874
EFest (%)	74.43 (8.37)	77.36 (8.88)	.065

Data are presented as mean (SD). Significant results are indicated in bold italic.

6MWT = six-minute walk test, CI = cardiac index, CO = cardiac output, CTI = contractility index, EDV = end-diastolic volume, EDFR = early diastolic filling ratio, EFest = estimated ejection fraction, HR = heart rate, LCWi = left cardiac work index, SD = standard deviation, SV = stroke volume, SVi = stroke volume index, SVR = systemic vascular resistance, SVRi = systemic vascular resistance index, VET = ventricular ejection time.

### 3.1. Adverse events

No adverse events occurred during the entire trial process.

## 4. Discussion

Weight-bearing Liuzijue Qigong training is an aerobic training method that combines respiratory training, resistance training, and mindfulness training for emotional regulation. The results confirmed our speculation that weight-bearing Liuzijue Qigong training would improve the pulmonary ventilation capacity of participants and the cardiac hemodynamic parameters of participants in both the resting and exercise stages.

The first mention of Liuzijue was in the ‘ Yangxing Yanminglu ‘ of Tao Hongjing in the Northern and Southern Dynasties. Since then, further records about Liuzijue Qigong have been produced. Before the Ming Dynasty, Liuzijue Qigong did not cooperate with body movements but was simply a Kung fu breathing exercise. Since the Ming Dynasty, Liuzijue Qigong began to acquire body movements, combining breathing with body guidance. The current developed form of Liuzijue Qigong is a relatively complete system. The theory underlying its techniques maintains the main framework of the 5-element and 5-Zang organ theory of Traditional Chinese Medicine. In principle, the guidance of the mind and the guidance of body movement follow the law of meridian circulation of Traditional Chinese Medicine.^[23]^

According to Liuzijue Qigong, the 6 words “Xu, He, Hu, Si, Chui, Xi” correspond to the 5 Zang organs of the human body. The connection function of its affiliated meridians is associated with 6 organs: the liver, heart, spleen, lung, kidney, and gallbladder; and is related to the body, acupoints, and emotional parts of the human body, with the aim of exercising the Zang organs, regulating qi and blood, and nourishing lung qi and other functions.^[24]^

There have been studies on the use of Liuzijue Qigong in the rehabilitation of lung function. Liu et al^[25]^ conducted a Liuzijue Qigong exercise and acupressure rehabilitation program for hospitalized patients with severe COVID-19,which showed that this training improves lung function and symptoms, such as dyspnea and cough, in patients, and shortened the length of their hospital stay. Wang et al^[26]^ carried out a systematic meta-analysis on the recovery of patients with COVID-19 treated using Liuzijue Qigong exercise and obtained supporting data. Wu et al^[2]^ showed that Liuzijue Qigong exercise could improve lung ventilation in patients with COPD, and the FEV1% increased significantly after training. Li et al^[7]^ demonstrated that long-term home-based Liuzijue exercise combined with clinical guidance can effectively improve the pulmonary function, exercise capacity, and quality of life of elderly patients with moderate to severe COPD.

The above-mentioned studies support the findings of the present study, that the participants’ FEV1 and FEV1/FVC were significantly improved after weight-bearing Liuzijue Qigong training. The MMEF and MVV of the participants also improved significantly, which might indicate that Liuzijue Qigong training improved small airway ventilation function and ventilation reserve capacity in healthy volunteers. One possible reason is that weight-bearing Liuzijue Qigong training not only includes respiratory exercise, but also operates in tandem with upper limbs exercise to further strengthen the role of the respiratory muscles. Previous studies have shown that upper limb resistance training can reduce dyspnea,^[27]^ effectively improve the inspiratory muscle strength, and increase the submaximal exercise capacity in patients with COPD.^[28]^ Studies by Benton and Wagner^[29]^ and Ramos et al^[30]^ also showed that upper limb resistance exercise helps to reduce the symptoms of COPD, improves cardiopulmonary capacity, and increases muscle mass and peripheral muscle strength more effective than isolated aerobic exercise. Using Liuzijue Qigong, upper limb weight-bearing training can improve the training intensity of the participants and might have a more appropriate exercise effect on healthy participants. In addition, the sounds of Liuzijue are generated by the exhaled airflow through 6 unique mouth forms. For example, in the “Xu” exercise, the mouth shape is designed to feel the airflow through the teeth, with the 2 lips parted, which increases airway pressure and reduces airflow restriction.^[31]^ Different modes of breathing training are carried out alternately, and thus the respiratory muscles might be trained more comprehensively. The combination of resistance training and traditional Qigong training might achieve better improvement in cardiopulmonary function. In further analysis of the MMEF, we found that the improvement in the MMEF might result mainly from the significant improvement of FEF50%, while FEF25% and FEF75% showed no significant changes. This suggested that the improvement in pulmonary ventilation function by weight-bearing Liuzijue Qigong training might be mainly reflected in the increase of expiratory flow rate in the middle of the lung, that is, the forced expiratory flow at 50% of FVC. The lack of a significant change in FEF75% might be because the participants were healthy volunteers without cardiopulmonary dysfunction who already had an excellent FEF75%. Therefore, although their FEF75% improved somewhat, the difference was not statistically significant.

Our study did not find significant changes in the VC and FVC measures, which represent lung volume. In addition, no associated changes were found in previous studies. This might be a simple validation of the Liuzijue Qigong training used in COPD patients without further aggravating emphysema. Of course, the final results need to be further confirmed by more specific and accurate experiments.

The possible mechanism of the effect is Liuzijue Qigong regulates rhythm of the breathing and aspiration, increases the breath depth and cycle, and trying to reach a “deep, long, and balance” target. Its diaphragmatic breathing may produce increased asynchronous and paradoxical breathing movements, and its prolonged expiration and slowing of the breathing rate produces a satisfactory effect.^[31]^ On the other hand, the weight-bear Liuzijue Qigong may exerted beneficial effects by improving respiratory muscle strength, peripheral skeletal muscle function and exercise capacity, reducing oxygen consumption, decreasing responses to hypoxic and hypercapnic conditions with better blood oxygenation without increasing minute ventilation,^[32]^ and decreasing the resting heart rate and sympathetic reactivity.^[33]^

The diaphragm is the major inspiratory pump muscle, and its neural activation generates a transdiaphragmatic pressure that drives air into the lungs. The diaphragm provides 75% of the increase in lung volume during quiet inspiration.^[34]^ Tang et al^[35]^ reported that the diaphragm movement in deep breathing of patients with COVID-19 increased significantly after 4 weeks of intervention. In this study, we found different results: the participants had a significant increase in DE during calm breathing and DCS during deep breathing after weight-bearing Liuzijue Qigong exercise. The DE during deep breathing and the DCS during calm breathing showed no significant changes. The possible reason is that calm breathing does not require rapid mobilization of the diaphragm and during deep breathing, the diaphragm amplitude in healthy people has reached a higher level, thus the change was not significant.

The results of 6MWT and cardiac hemodynamic parameters were interesting. Our findings showed that the distance traveled in the 6MWT of participants increased significantly and their cardiac hemodynamic parameters improved considerably after weight-bearing Liuzijue Qigong exercise. In the resting stage, we found that although the HR did not change significantly, the SV tended to increase, and the CO and CI, which are representative of cardiac function, were significantly improved. In addition, the CTI and EFest of the participants also improved significantly during the resting stage. These are cardiac dynamic parameters related to CO. In the walking stage, we found significant improvement in the SVi and HR, which are 2 indicators that directly affect the CO and CI. Moreover, the left cardiac work index also improved significantly. Although there was no significant difference in EFest during the walking stage, the parameter did increase. This might be related to the fact that our participants are healthy adults. They have good cardiac ejection function and thus we might need more participants to significantly reflect the improvement of this index. After training with weight-bearing Liuzijue Qigong, we also found that the SVR and SVRi of participants were significantly reduced both in the resting stage and the walking stage. Another interesting and important finding is that after training, the EDFR of participants in the resting stage was significantly improved compared with that before training. The reasons for these results might be multifaceted. First, weight-bearing Liuzijue Qigong training is an aerobic training that combines respiratory training and upper limb resistance training. The training process has a corresponding exercise effect on cardiac function. Kawecka-Jaszcz et al^[36]^ conducted slow breathing training in patients with chronic heart failure and concluded that slow breathing training can improve physical capacity and systolic heart function. Cordina et al^[37]^ identified that resistance muscle training improved muscle mass and strength, and was associated with improved cardiac filling, stroke volume, and exercise capacity. Moriki et al^[38]^ also showed that aerobic training improved SV and CO. Second, the Liuzijue Qigong training process needs a relaxed mood, which is a mind-body therapy. A previous study demonstrated that emotional relaxation training can reduce the excitability of sympathetic nerves and might make the heart more fully relaxed.^[39]^ In this study, the increase in EDFR might have been be mainly caused by regulation of autonomic nerves. In addition, a previous study showed that inspiratory training increases intrathoracic negative pressure and increases the venous return by 40%.^[37]^ They explained that resistance training augmentation of peripheral muscle bulk promotes venous return. This also supports the increased EDFR in our study.

Data from the WHO show that about 3.2 million people die because of an unhealthy lifestyle and lack of proper exercise every year. Lee et al^[40]^ also proposed that 6–10% of all deaths from non-communicable diseases can be attributed to a lack of physical activity. The current lack of physical activity has become the fourth leading cause of death globally.^[41]^ Moderate physical activity is positively associated with cardiorespiratory fitness.^[42,43]^ However, people lacked the opportunity for outdoor activities during the COVID-19 epidemic. According to a multinational survey with 13503 participants from 14 countries, individual movement at both moderate and vigorous intensity levels decreased by more than 40% during lockdowns.^[44]^ Another survey from Spain also got the same result.^[45]^ Weight-bearing Liuzijue Qigong training, executed regularly, could be an option to protect health both during a pandemic and in normal life. Weight-bearing Liuzijue Qigong training is a simple and convenient cardiopulmonary training method that could be carried out at home or in the workplace. The training does not need a professional venue, equipment, or even a large space, which could support its clinical application and popularization.

Previous studies mostly used Liuzijue Qigong training for patients with respiratory diseases, and there is almost no research about the effect of Liuzijue Qigong on cardiovascular function. The innovation of this study is we focused on weight-bearing Liuzijue Qigong training, which is more suitable to improve not only the pulmonary function, but also the cardiovascular function of healthy people. It can be used as a home cardiopulmonary training method actively selected by clinicians to improve the cardiopulmonary function of healthy people or sub-healthy people, for the researchers, further studies on its cardiovascular effects may be a key point

### 4.1. Limitations

This study had some limitations. First, our participants were only trained for 4 weeks, and there was a lack of long-term follow-up. Second, the sample size of this study was 12 cases, which is relatively small. However, the results were produced and analyzed quickly, allowing us to publicize this home exercise method as soon as possible, especially during the pandemic. In addition to independent repetition, a study with a larger sample size is required to validate our findings, which will be the subject of our subsequent research. Moreover, we only studied the effect of weight-bearing Liuzijue Qigong training on healthy people, and in future research, we will focus of the effect of weight-bearing Liuzijue Qigong training on other patients, such as those with osteoarticular diseases or nervous system diseases.

## 5. Conclusion

The purpose of this study was to assess Liuzijue Qigong training as a convenient, simple, at-home training method for people who lack the opportunity for outdoor activities, to improve or maintain their cardiopulmonary function. We proved that weight-bearing Liuzijue Qigong training, as an aerobic training method, combines respiratory training, resistance training, and mindfulness training for emotional regulation, and could improve cardiopulmonary function in healthy volunteers.

## Author contributions

**Conceptualization:** Desheng Li, Mei Shen, Xiaoyan Yang.

Data curation: Desheng Li.

Formal analysis: Desheng Li.

Investigation: Chunxiu Zhou.

Methodology: Desheng Li, Mei Shen, Xiaoyan Yang.

Supervision: Desheng Chen.

Visualization: Desheng Li, Desheng Chen.

Writing – original draft: Desheng Li.

Writing – review & editing: Mei Shen, Qiuyang Qian.
